# Supplementary material for: Machine learning-coupled combinatorial mutagenesis enables resource-efficient engineering of CRISPR-Cas9 genome editor activities
Source: Nat Commun. 2022 Apr 25;13:2219. doi: 10.1038/s41467-022-29874-5 (PMC9039034; doi:10.1038/s41467-022-29874-5)
Supplement: Supplementary file 1 — Supplementary Information [file 41467_2022_29874_MOESM1_ESM.docx]

**Supplementary Figure 1. Generation of training datasets with increased sequence diversity.** Bar charts show the relative abundance of N-distance neighbors in the diverse versus random dataset of SpCas9’s on-target activities with Sg5 and Sg8, as a proxy to the sequence diversity of the generated datasets that represent 5, 10, 20 and 50% of the empirical data. As implemented by our filtering scheme (see Methods for details), the relative abundance of neighbours carrying 1 mismatch and 2 mismatches were reduced and there was an increase in the neighbours carrying 7 and 8 mismatches in the diverse dataset when compared to the random dataset with the same size of 5, 10, and 20%. The data presented here represent the averaged values from three replicate training datasets of the same percentage of diversified and randomized selection scheme.**
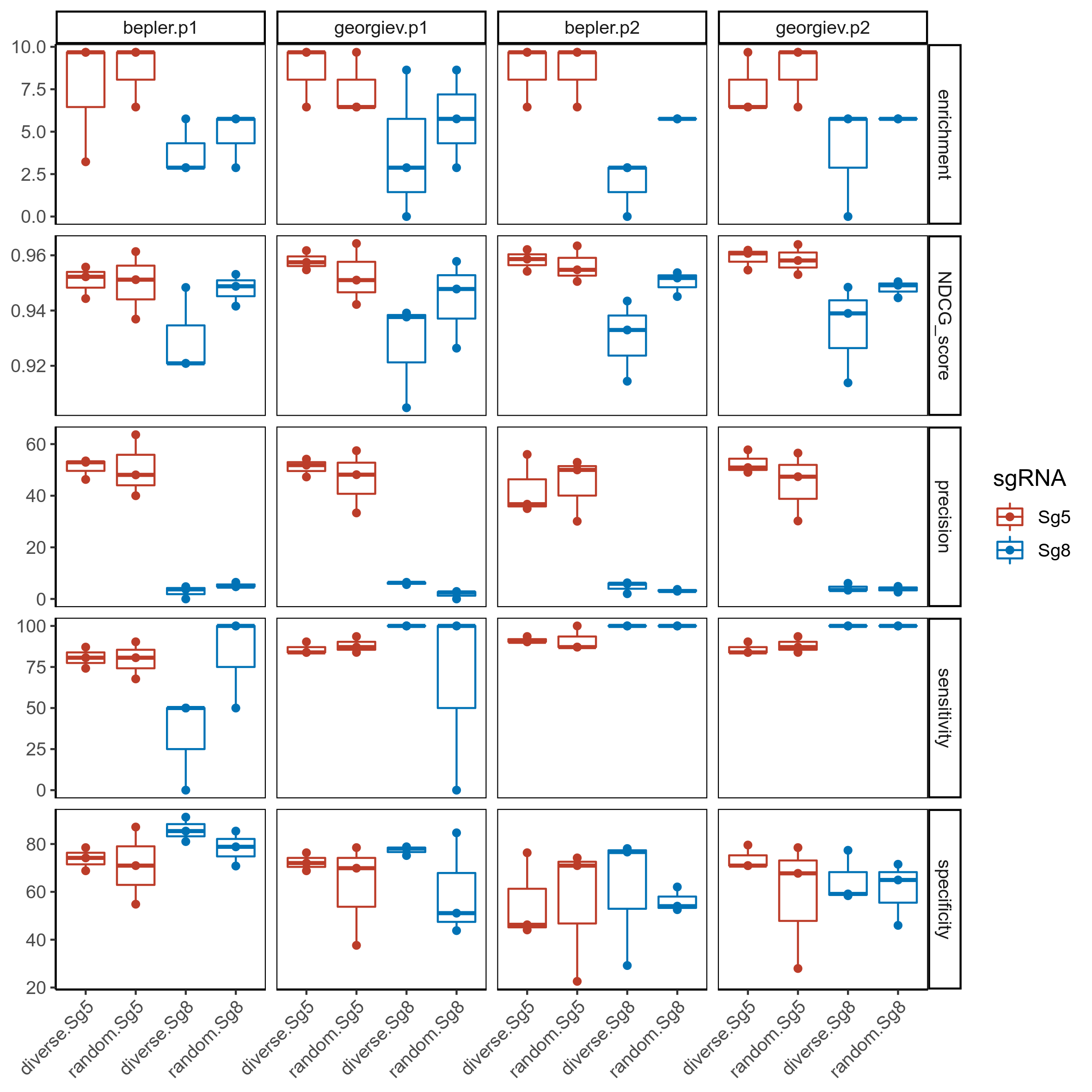
**

**Supplementary Figure 2. Performance of MLDE on SpCas9 activity predictions using different embeddings, model parameters, and input datatypes.** Metrics that measure the performance of MLDE models with Bepler/Georgiev embedding combined with more complex neural networks models (parameter 1 - p1) or with an ensemble of more simple models such as random forests and SVM (parameter 2 - p2) include (from top to bottom rows) enrichment (ranges from 0 to infinity), NDCG (ranges from 0 to 1), precision (ranges from 0 to 100%), sensitivity (ranges from 0 to 100%), and specificity (ranges from 0 to 100%) on SpCas9 on-target activity with Sg5 (red) and Sg8 (blue) using random or diverse training data that correspond to 5%, 10%, 20% of the SpCas9 variant library (n=3 independent MLDE runs for each data size). MLDE performance was evaluated using the test set variants that correspond to 20% of the library that were withheld from the training data. The box indicates the 25 (bottom), 50 (central) and 75 (top bar) percentiles of the group, whiskers extend to 1.5 times the interquartile range.

**
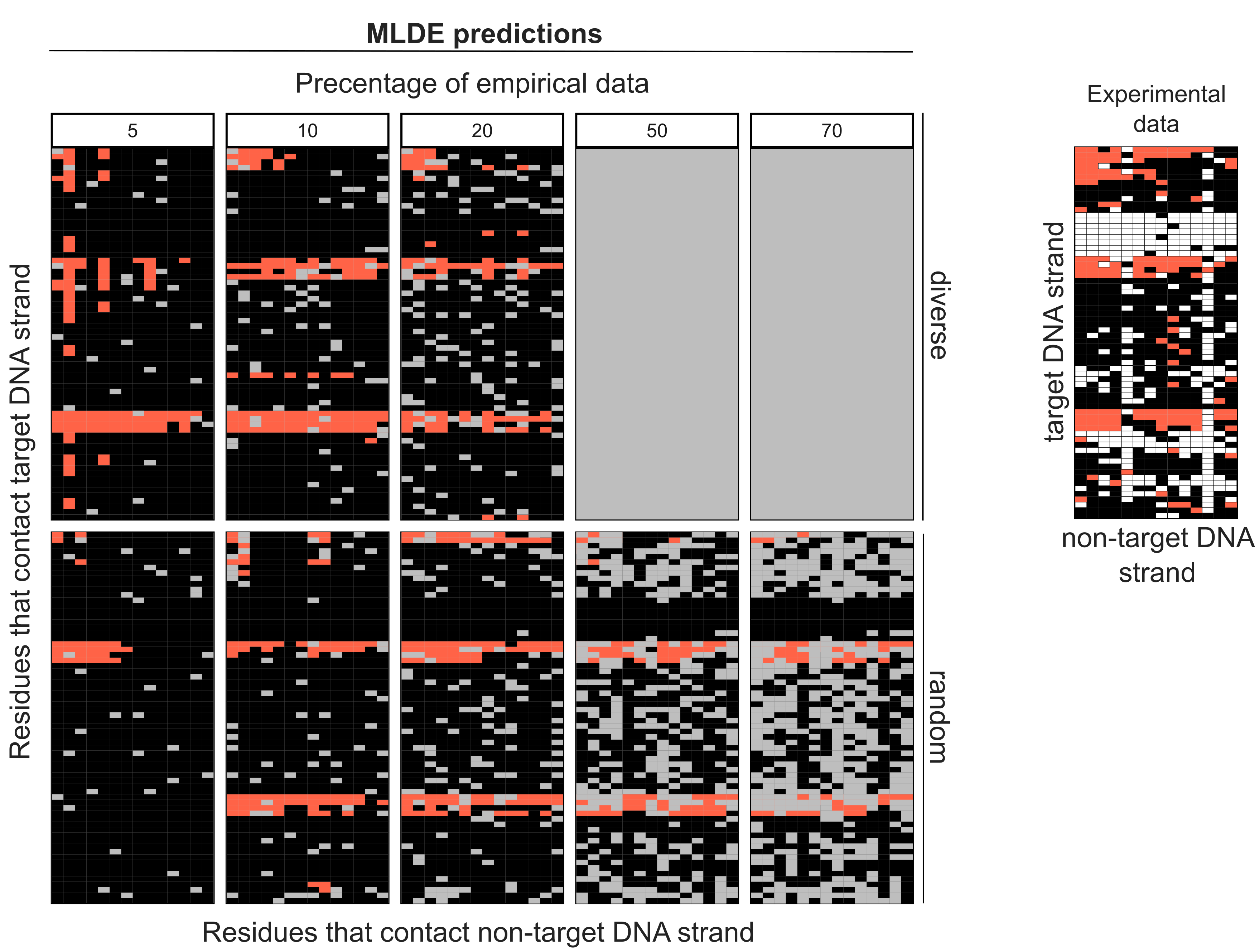
**

**Supplementary Figure 3. Prediction of top-performing variants of SpCas9 with at least 70% of wild-type activity using MLDE.** Plots showing the predictions yielded by the top-performing runs of MLDE (Bepler embedding + network model (parameter 1)) on diversified (top panel) and randomized (bottom panel) training data of different size (5, 10 and 20%) for Sg5 on-target activity scores. Top-performing MLDE runs using the Bepler embedding and modelling parameter 1 on randomized training data of size 50 and 70% are shown in the bottom panels. MLDE run was not carried out on diverse training data of size 50 and 70% because of the lack of dissimilarities among variants compared to random selection (see Supplementary Figure 1). The empirical Sg5 on-target activity dataset is shown at the right panel. Missing empirical data is highlighted in white. Variants are arranged according to the target-strand (y-axis) and non-target-strand (x-axis) sequences. Only test data are shown in the plot; training data that are removed are highlighted in grey. Variants that are predicted with comparable wild-type activity are highlighted in tomato color whereas those possesses lower than 70% wild-type activity are filled in black. Source data are provided as a Source Data file.**
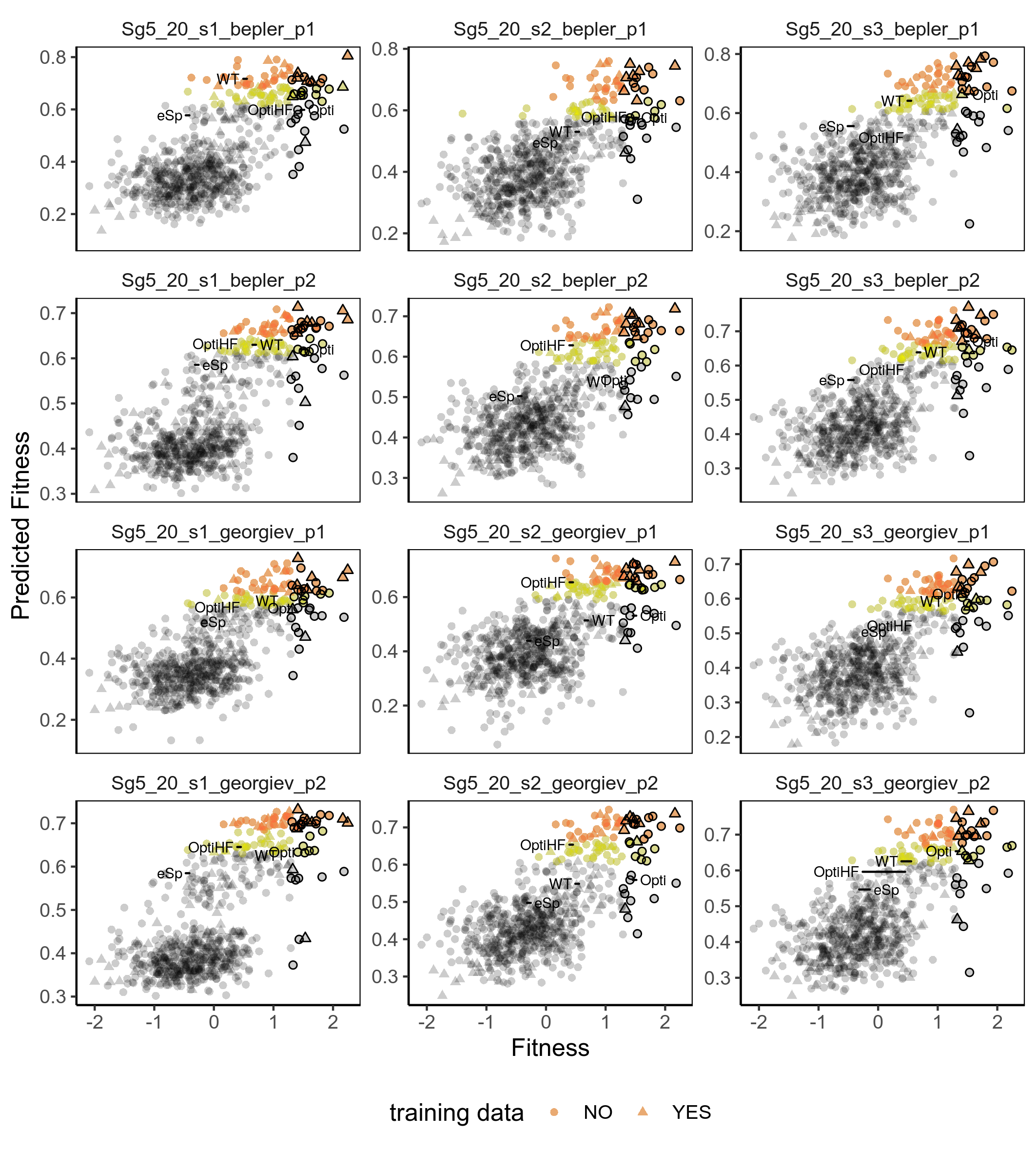
**

**Supplementary Figure 4. MLDE runs on Sg5 on-target activity with SpCas9.** Predicted versus empirical fitness of variants in MLDE runs using 20% input training data, given the different combinations of embeddings (Bepler/ Georgiev) and models (p1 and p2). The predicted fitness by MLDE is plotted against the empirical fitness of the Sg5 on-target activity in three independent runs (i.e., s1, s2, and s3) ranked according the NDCG and enrichment score). The top 1-5% and 5-10% hits in the prediction are highlighted in orange and yellow respectively, while the top 5% variants from the empirical data are outlined in black. Wild-type SpCas9 and other previously characterized variants (eSpCas9, Opti-SpCas9 and OptiHF-SpCas9) are labelled. Source data are provided as a Source Data file.

**
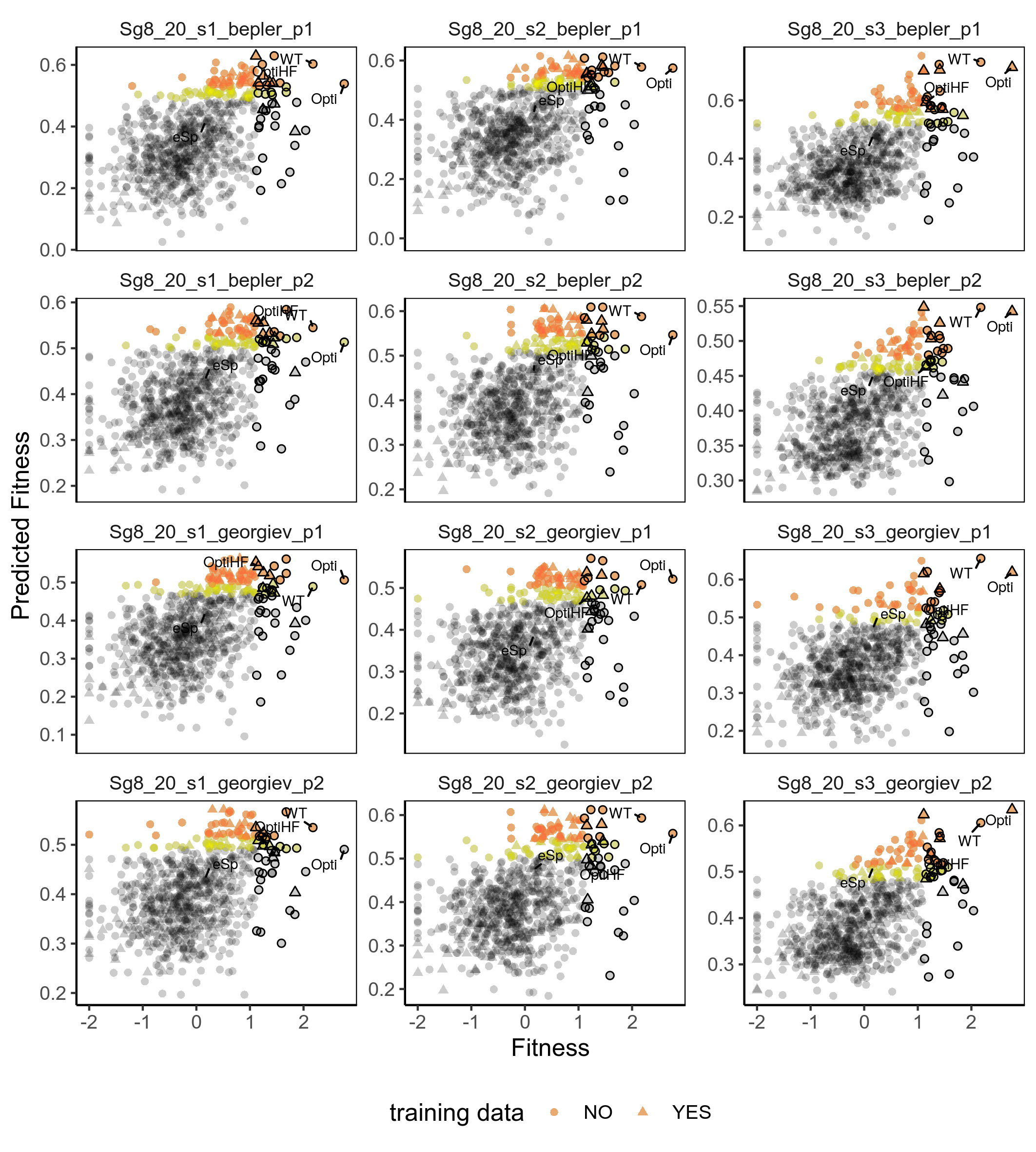
**

**Supplementary Figure 5. MLDE runs on Sg8 on-target activity with SpCas9.** Predicted versus empirical fitness of variants in MLDE runs using 20% input training data, given the different combinations of embeddings (Bepler/Georgiev) and models (p1 and p2). The predicted fitness by MLDE is plotted against the empirical fitness of the Sg8 on-target activity in three independent runs (i.e., s1, s2, and s3) ranked according the NDCG and enrichment score). The top 1-5% and 5-10% hits in the prediction are highlighted in orange and yellow respectively, while the top 5% variants from the empirical data are outlined in black. Wild-type SpCas9 and other previously characterized variants (eSpCas9, Opti-SpCas9 and OptiHF-SpCas9) are labelled. Source data are provided as a Source Data file.

**
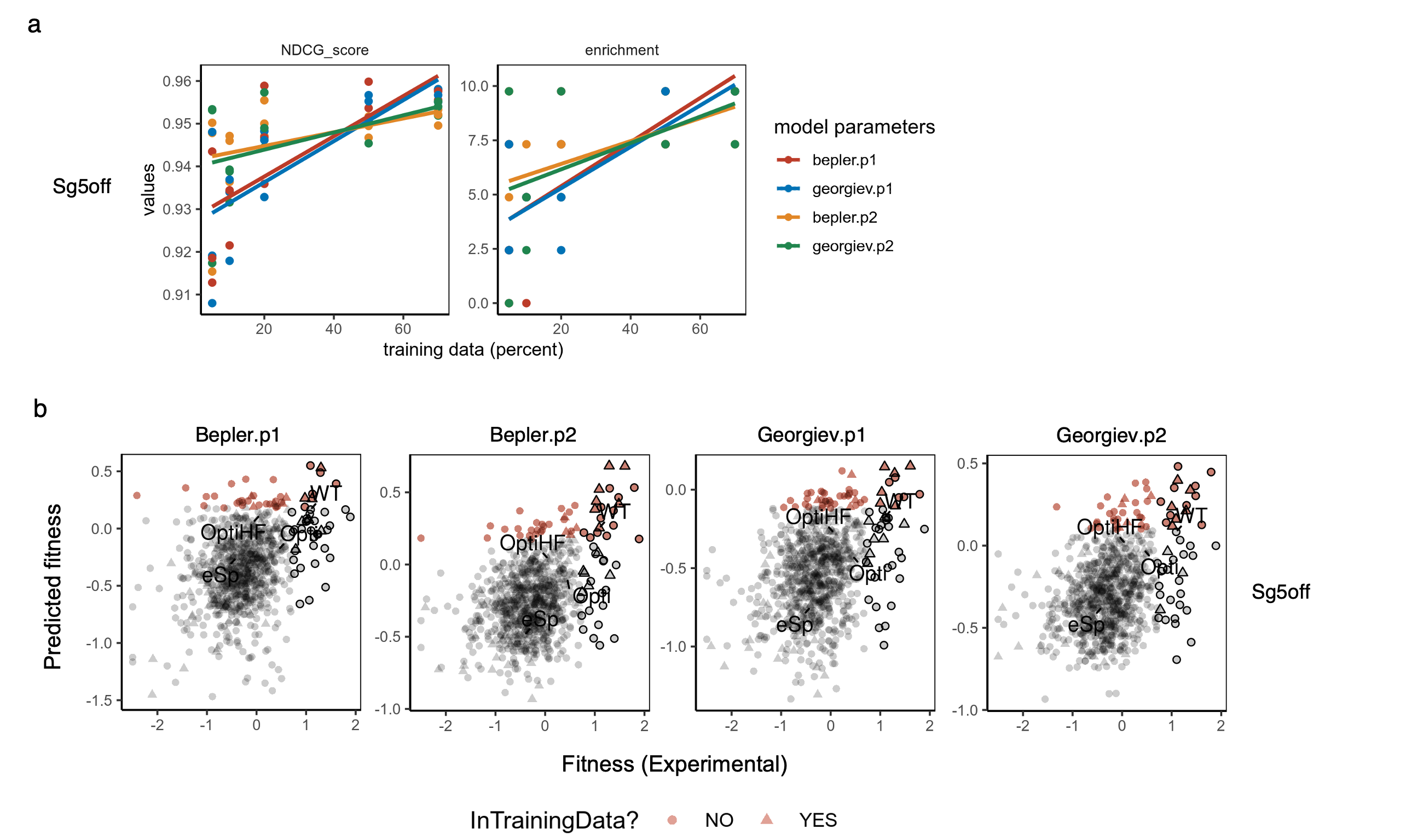
**

**Supplementary Figure 6. Performance of MLDE predictions on Sg5 off-target activity with SpCas9.** **a,** NDCG score and Enrichment of MLDE runs using a combination of embeddings (Bepler/ Georgiev) and models (p1 and p2) are plotted against the size of training data. The best fit line summarizes 3 replicates for each embedding and model parameter combination of MLDE runs using 5, 10, 20, 50 and 70% of training data. **b,** Predicted versus empirical fitness of variants in the best-performing MLDE runs using 20% input training data, given the different combinations of embeddings and model parameters. The predicted fitness by MLDE is plotted against the empirical fitness of the Sg5 off-target activity in the best-performing runs. The top 5% hits in the prediction are highlighted in red, while the top 5% variants from the empirical data are outlined in black. Wild-type SpCas9 and other previously characterized variants (eSpCas9, Opti-SpCas9 and OptiHF-SpCas9) are labelled. Source data are provided as a Source Data file.

**
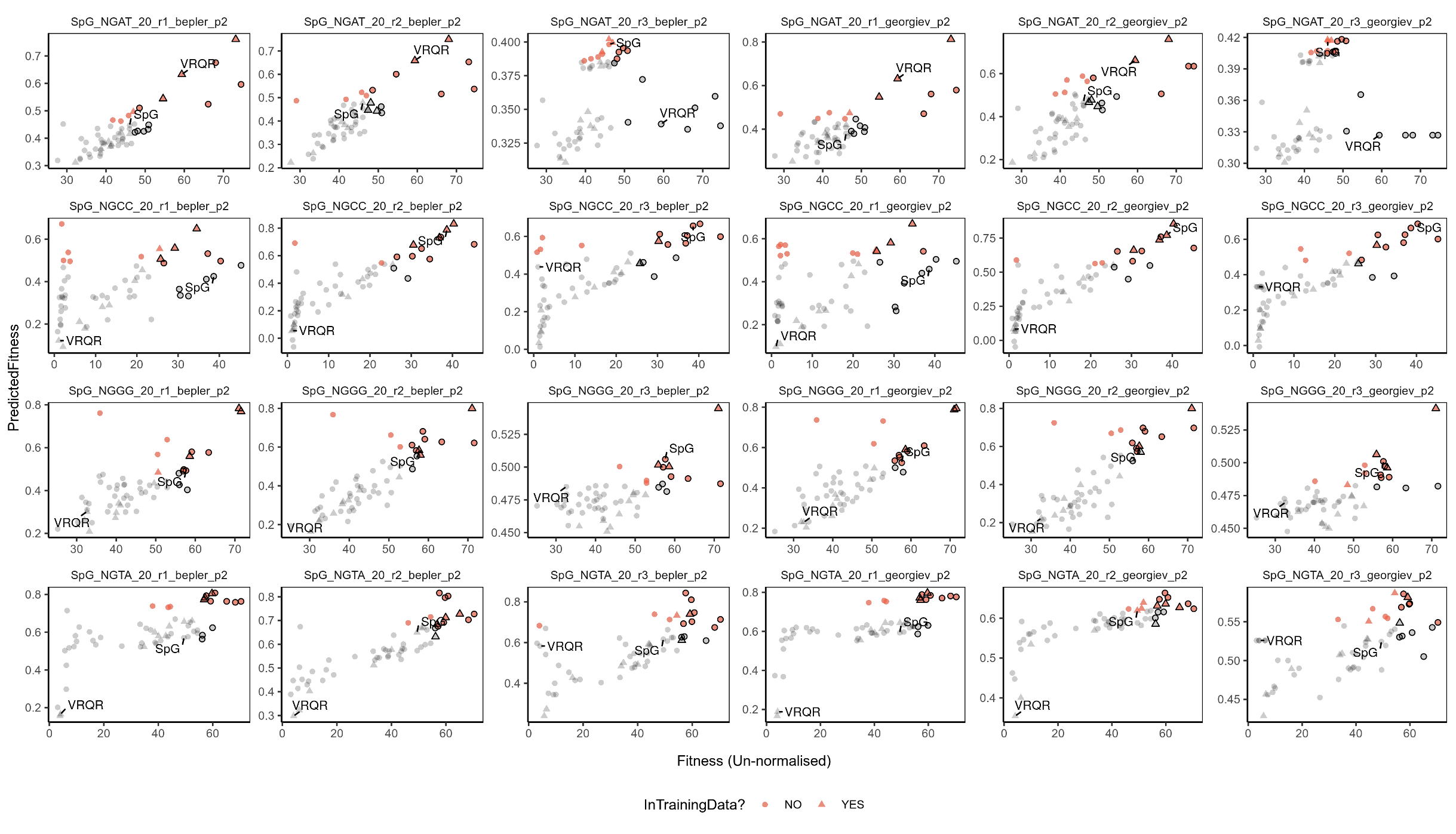
**

**Supplementary Figure 7. MLDE runs on SpCas9’s activities at NGN PAMs.** Predicted versus empirical fitness of variants in MLDE runs using 20% input training data, given the different combinations of embeddings (Bepler/ Georgiev) and model (p2). The predicted fitness by MLDE is plotted against the empirical fitness of the SpCas9’s activity on NGN PAMs (NGAT, NGCC, NGGG and NGTA) in three independent runs (i.e., r1, r2, and r3). The top 20% (12 variants) in the prediction are highlighted in red and the top 20% from the empirical data outlined in black. The parental variant, VRQR, and the variant SpG are labelled. Source data are provided as a Source Data file.

**
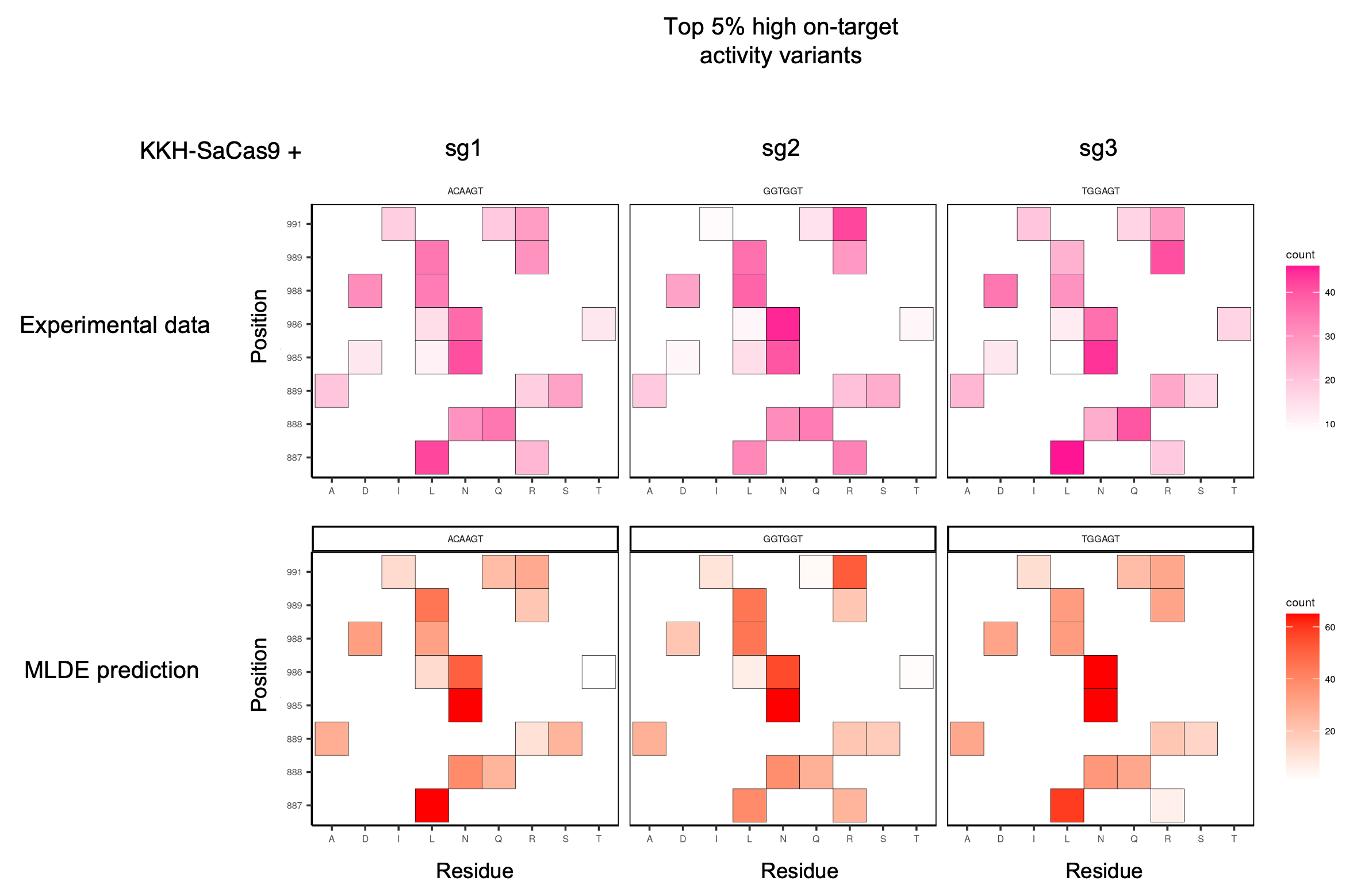
**

**Supplementary Figure 8. Comparison of MLDE prediction results and experimental screen data over KKH-SaCas9 variants with top 5% activities in the screening library.** Heatmaps showing the occurrences (counts) of the amino-acid residues per site among the top 5% variants identified in the experimental (top panels) screens and the best MLDE runs (bottom panels) using Georgiev embedding and modelling parameter 2. Three independent sgRNAs (sg1, sg2, sg3) were used.


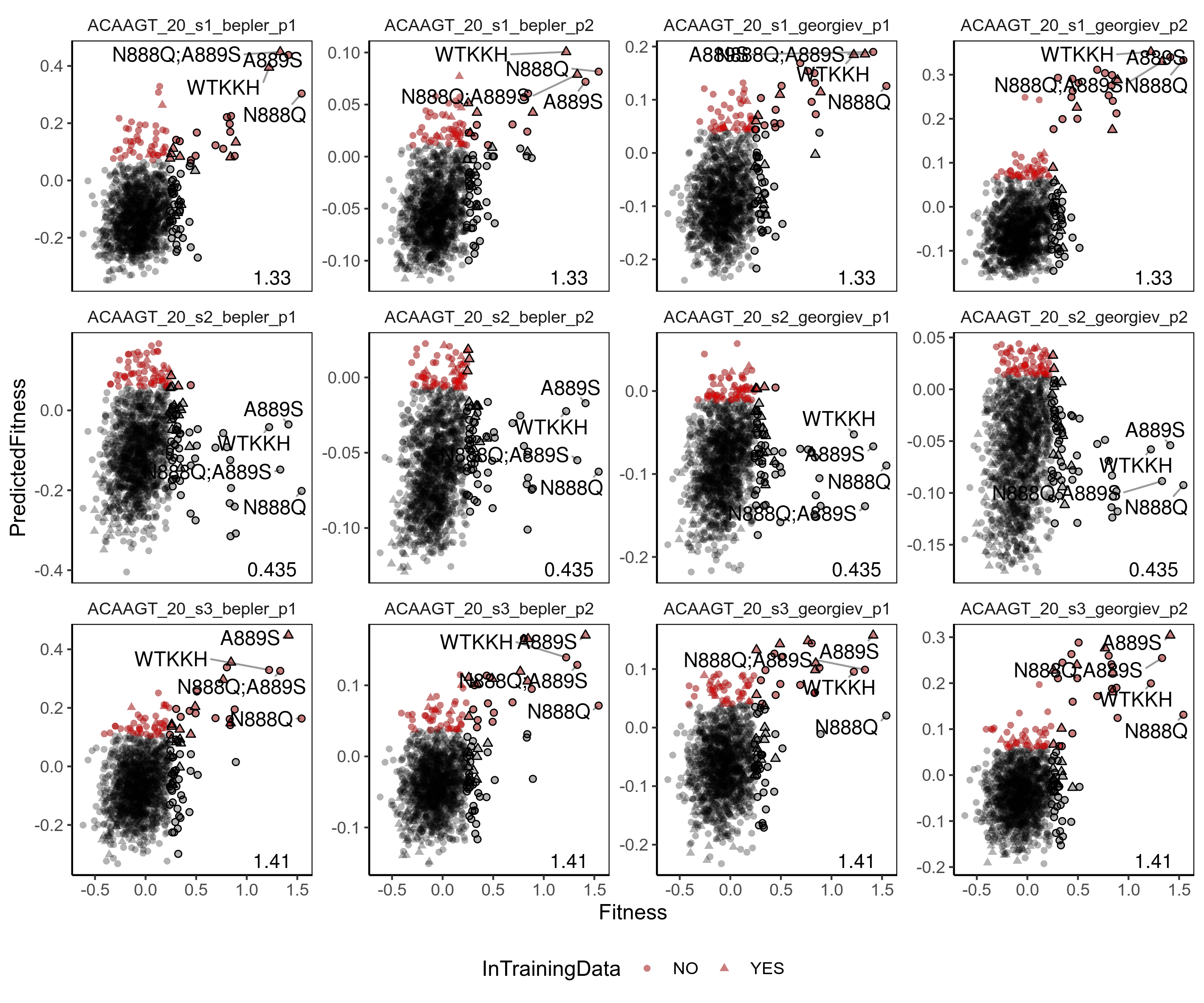


**Supplementary Figure 9. MLDE runs on sg1 on-target activity with KKH-SaCas9.** Predicted versus empirical fitness of variants in three replicates of MLDE runs using 20% input training data and the different combinations of embeddings (Bepler/ Georgiev) and models (p1 and p2) for sg1 sgRNA (ACAAGT). The predicted fitness by MLDE is plotted (y-axis) against the empirical fitness data (x-axis). The values of maximum fitness in the training data is indicated at the bottom right corner of each panel. The top 5% hits in the prediction are highlighted in red, while the top 5% variants from the empirical data are outlined in black. Wild-type KKH SaCas9 (WT-KKH) and top-performing variants N888Q, N888Q/A889S, and A889S are labelled. Source data are provided as a Source Data file.

**
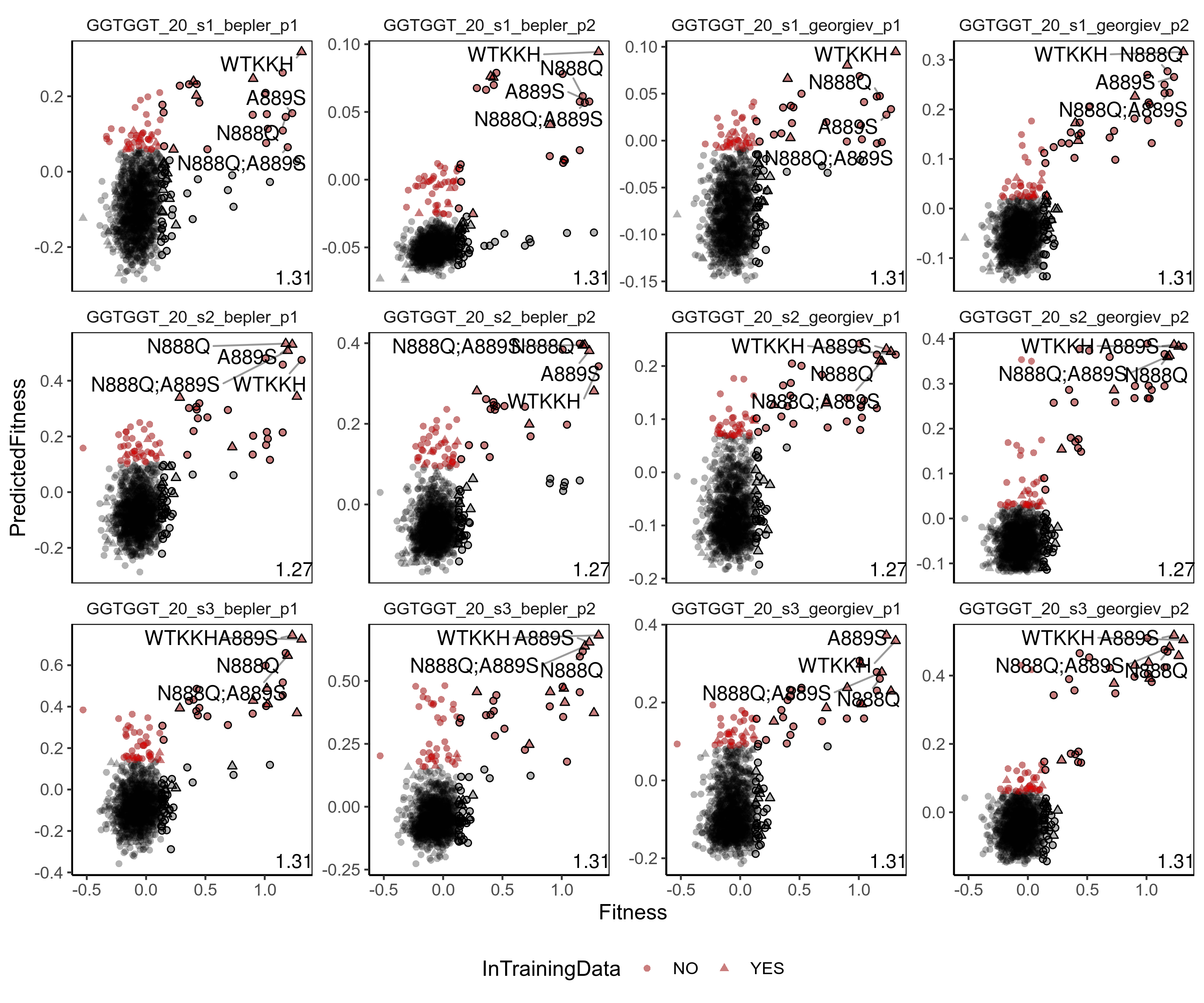
**

**Supplementary Figure 10. MLDE runs on sg2 on-target activity with KKH-SaCas9.** Predicted versus empirical fitness of variants in three replicates of MLDE runs using 20% input training data and the different combinations of embeddings (Bepler/ Georgiev) and models (p1 and p2) for sg2 sgRNA (GGTGGT). The predicted fitness by MLDE is plotted (y-axis) against the empirical fitness data (x-axis). The values of maximum fitness in the training data is indicated at the bottom right corner of each panel. The top 5% hits in the prediction are highlighted in red, while the top 5% variants from the empirical data are outlined in black. Wild-type KKH SaCas9 (WT-KKH) and top-performing variants N888Q, N888Q/A889S, and A889S are labelled. Source data are provided as a Source Data file.

**
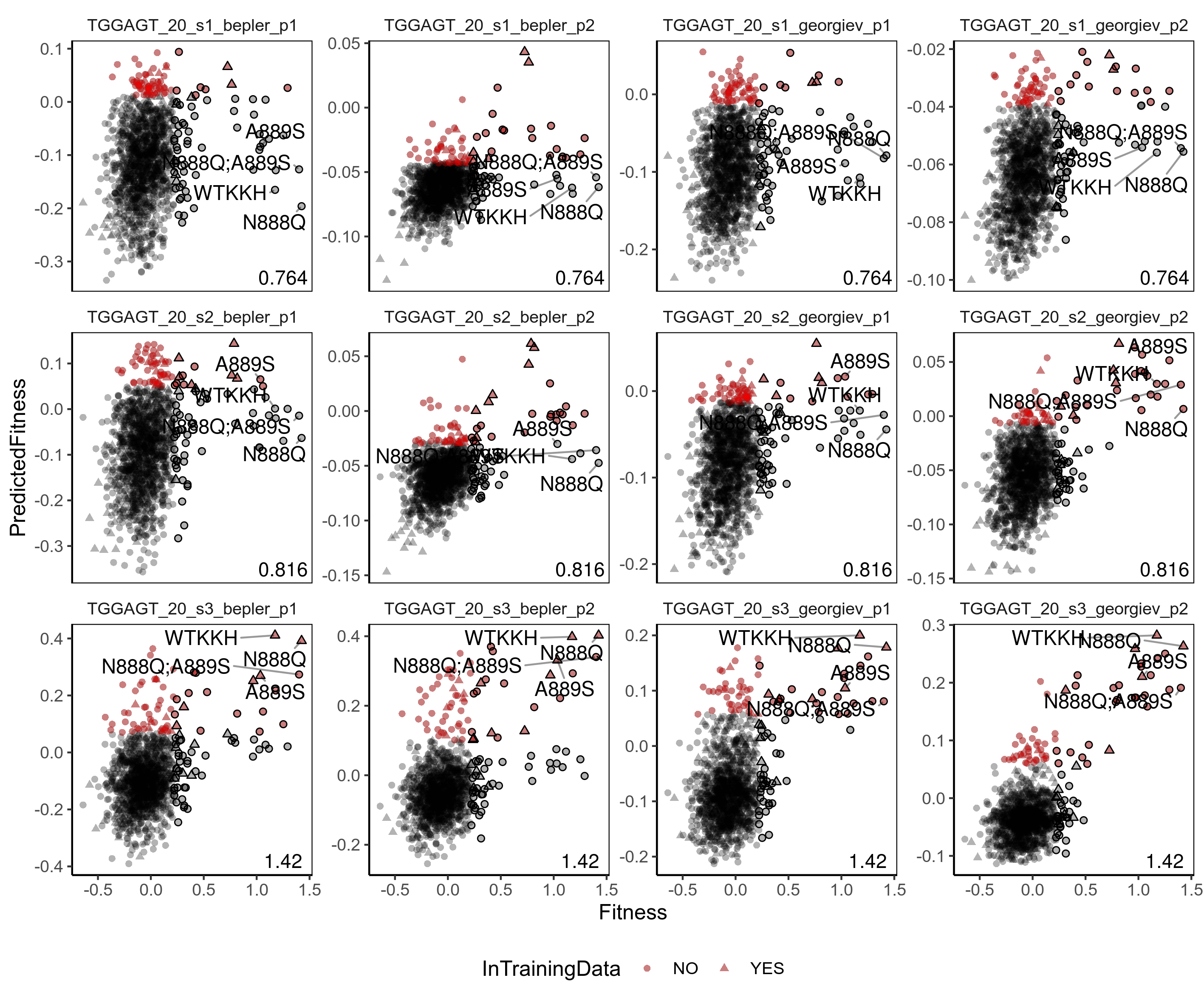
**

**Supplementary Figure 11. MLDE runs on sg3 on-target activity with KKH-SaCas9.** Predicted versus empirical fitness of variants in three replicates of MLDE runs using 20% input training data and the different combinations of embeddings (Bepler/ Georgiev) and models (p1 and p2) for sg2 sgRNA (TGGAGT). The predicted fitness by MLDE is plotted (y-axis) against the empirical fitness data (x-axis). The values of maximum fitness in the training data is indicated at the bottom right corner of each panel. The top 5% hits in the prediction are highlighted in red, while the top 5% variants from the empirical data are outlined in black. Wild-type KKH SaCas9 (WT-KKH) and top-performing variants N888Q, N888Q/A889S, and A889S are labelled. Source data are provided as a Source Data file.

GFP disruption (%)

GFP disruption (%)

Day 7

Day 12

GFP disruption (%)

sg3

sg2

sg1

N888Q

N888Q; A889S

A889S

WT (KKH)

**P*=0.003

**P*=0.046

53.0

54.6

65.5

57.9

73.7

76.2

81.9

77.0

54.7

54.9

64.3

62.8

75.8

74.8

81.7

78.3

39.8

36.1

36.5

34.0

61.2

63.4

62.9

62.6

**Supplementary Figure 12. Validation of the screen hits of activity-enhanced KKH-SaCas9 variants using non-pooled assays.** KKH-SaCas9 variants carrying mutations on residues 888 and/or 889 were individually constructed and characterized using GFP disruption assays with three sgRNAs. The editing efficiency of the KKH-SaCas9 variants was measured as the percentage of cells with depleted GFP fluorescence using flow cytometry. Mean editing efficiency +/- SD (error bar) obtained from n=8 biological independent samples are shown. Statistical significance was analyzed by one-way ANOVA with Tukey’s test. The *P* values of 0.003-0.046 indicate the comparisons with the wild type (WT) variant's activity at the same time point. Source data are provided as a Source Data file.

**Supplementary Figure 13. Molecular models of other tested variants with mutations introduced to residues 888 and 889 at the WED domain of SaCas9.** The dotted lines denote the interactions modelled among the amino-acid residues of SaCas9, as well as those modelled among the amino-acid residues of SaCas9 and the target DNA’s backbone.


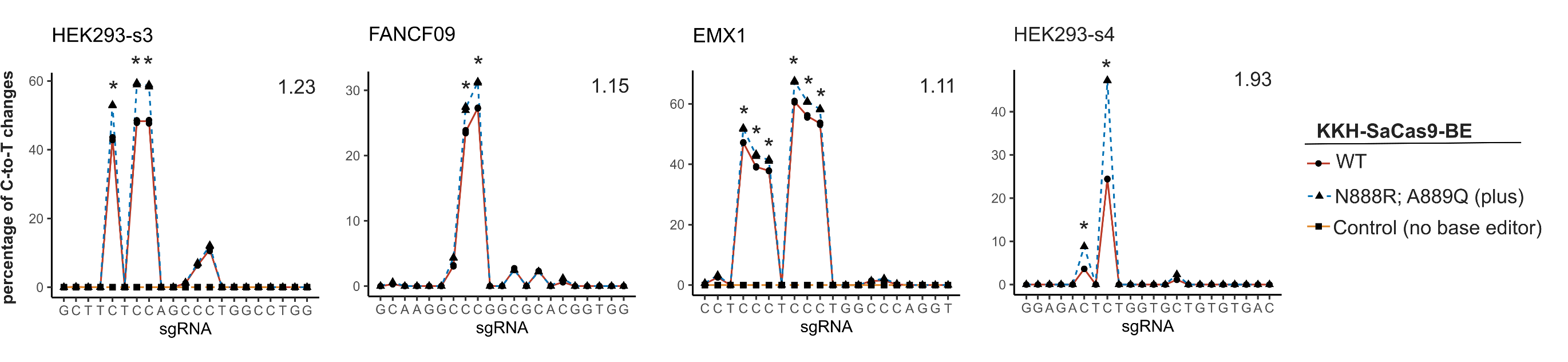


**Supplementary Figure 14.** **N888R/A889Q mutations increased the activity of KKH-SaCas9-derived cytosine base editor.** Cell expressing KKH-SaCas9-BE4max wild-type or its variant carrying N888R/A889Q were infected with lentiviruses encoding sgRNAs against the endogenous targets. The editing efficiency was measured using deep sequencing assay. Values reflect the mean of three replicates. Asterisks indicate the comparisons with the wild type variant’s activity (*P* < 0.05). The percentage of C-to-T changes for N888R/A889Q over wild-type at the most edited base in the target site is indicated at the top right corner of each panel. Source data are provided as a Source Data file.


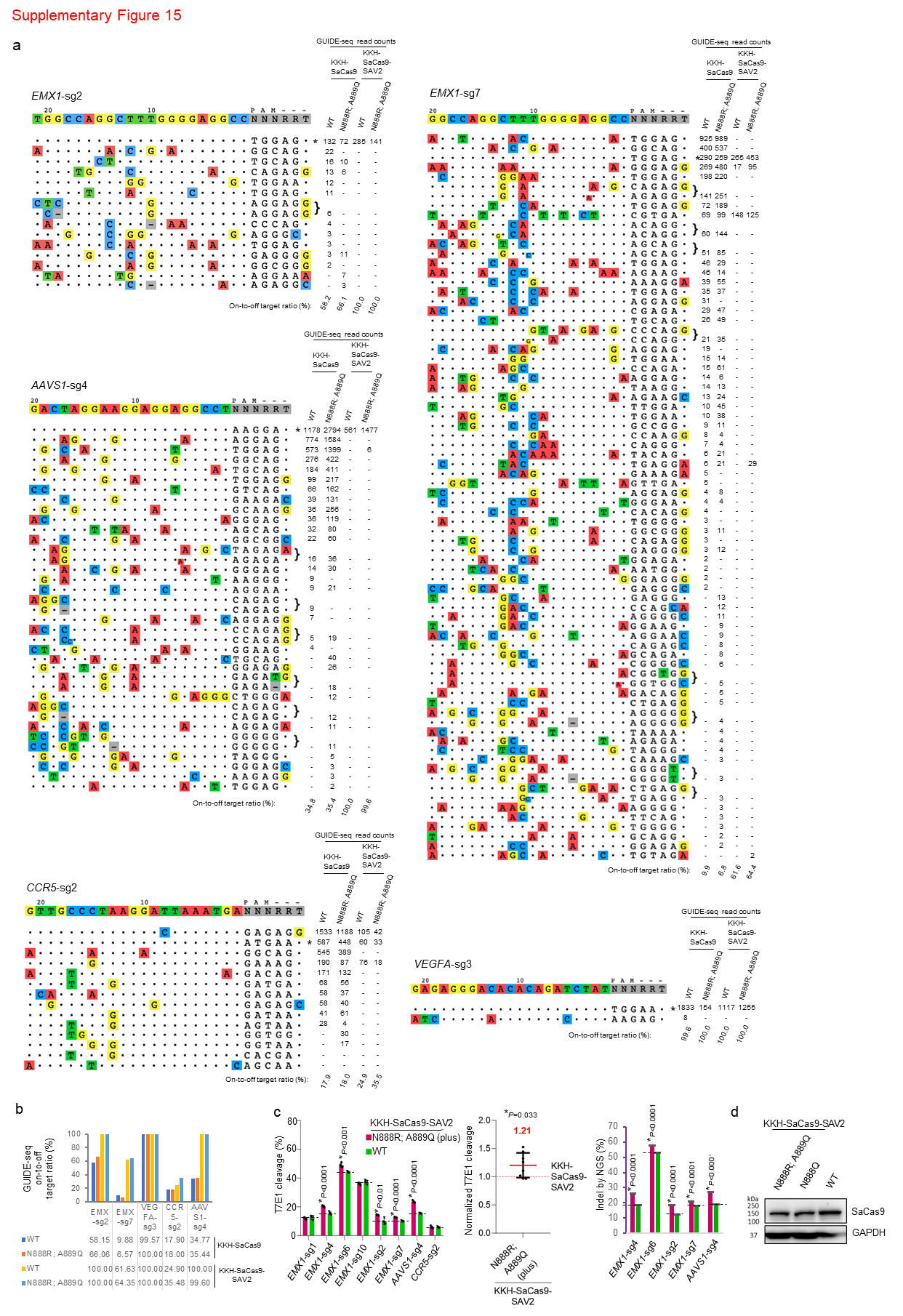


**Supplementary Figure 15. The activity-enhancing mutations increase activity of high-fidelity KKH-SaCas9-SAV2 variant while maintaining its high editing accuracy. a, b,** GUIDE-seq genome-wide specificity profiles for KKH-SaCas9 and KKH-SaCas9-SAV2 variants with or without N888R/A889Q mutations. The on-to-off target ratio presented in panel (b) were determined for each of the five independent sgRNAs used. The full dataset is presented in panel (a). Results for the controls without N888R/A889Q mutations were adapted from our published work ^16^ for easy side-by-side comparison. Mismatched positions in off-target sites are colored, and GUIDE-seq read counts were used as a measure of the cleavage efficiency at a given site. **c,** Assessment of high-fidelity KKH-SaCas9 variants’ on-target editing with sgRNAs targeting endogenous loci. The percentage of sites with indels was measured using a T7 endonuclease I (T7E1) assay (left panels) and deep sequencing assay (right panel). Mean editing efficiency +/- SD (error bar) are shown for each loci tested measured in n=4 and n=3 biological independent samples in (c) and (d), respectively. Statistical significance was analyzed by Student’s t-test. The *P* values of <0.001 indicate the comparisons with the wild type variant’s activity. The ratio of the on-target activity of KKH-SaCas9-SAV2 with N888R/A889Q mutations to the activity of KKH-SaCas9-SAV2 was determined, and the median for the normalized percentage of indel formation is shown and highlighted by a red line (n=8, one-sample t-test). **d,** Western blot analysis on protein expression of the KKH-SaCas9-SAV2 variants. Source data are provided as a Source Data file.

**
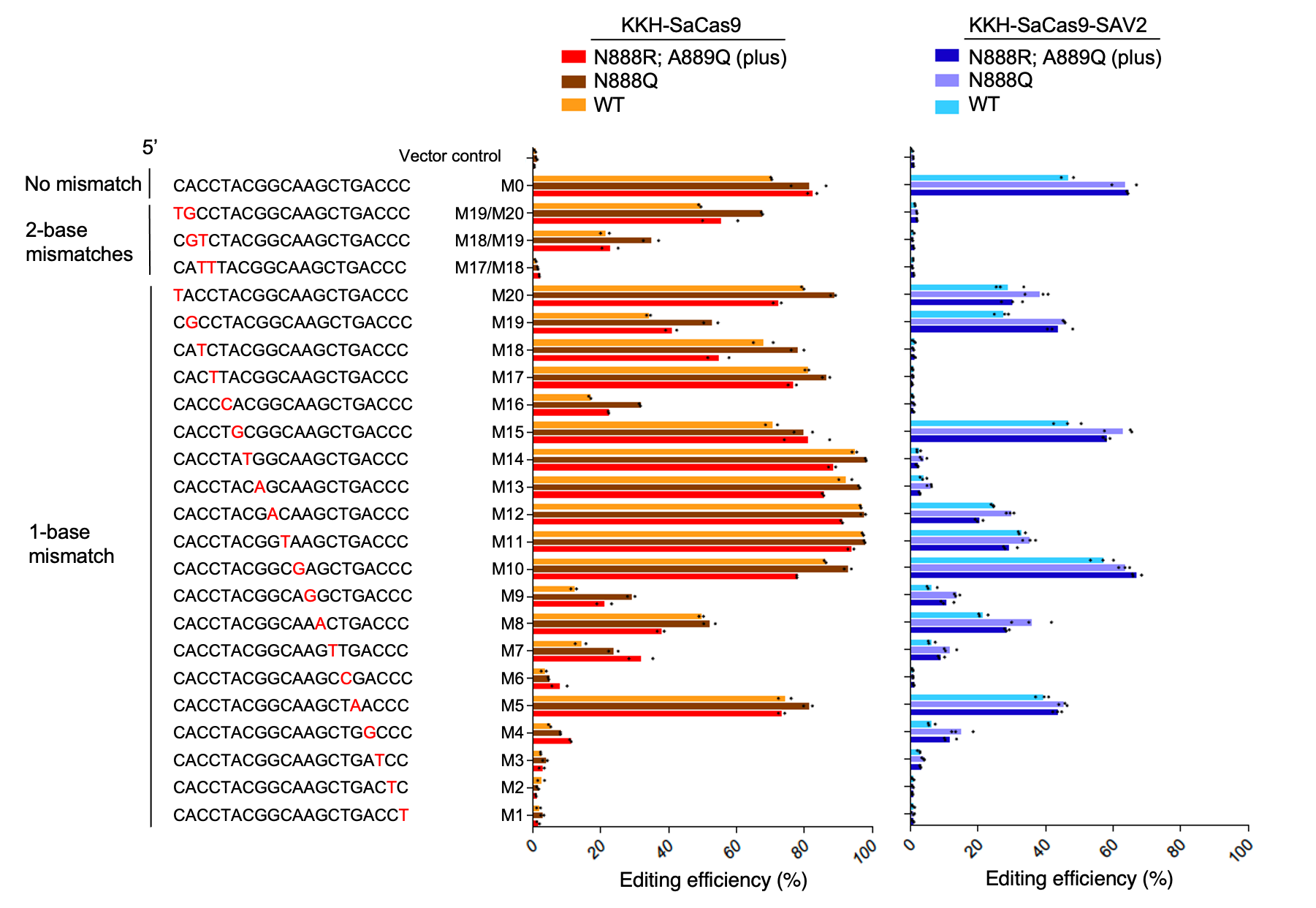
**

**Supplementary Figure 16. The activity-enhancing mutations increased activity of high-fidelity KKH-SaCas9-SAV2 variant and generated reduced off-target edits at sites harbouring sequences with single and double mismatch(es) to sgRNA spacer compared to wild-type.** Cells expressing the KKH-SaCas9 variants were infected with lentiviruses encoding sgRNAs carrying no or one- to two- base mismatch(es) against the target. The editing efficiency was measured as the percentage of cells with depleted GFP fluorescence using flow cytometry. Values reflect the mean of n=2 or 3 independent biological replicates. Source data are provided as a Source Data file.

**Supplementary Figure 17. Potential strategies of using MLDE to expand the number of mutation sites surveyed. a,** Multiple smaller focused libraries with mutagenesis up to 6 sites (highlighted in light blue) with 1-2 sites in common to another library are constructed. The empirical data of all 7 screens are combined and fed into MLDE to identify the best variants across all of the sites. **b,** Perform iterative rounds of targeted mutagenesis and MLDE. Up to 6 sites (highlighted in light blue), each with a few candidate residues selected from structure-guided design, are screened in a library. The top-performing variants predicted by MLDE from each round seeds the mutagenesis library of the next round with a new set of amino-acid sites subjected to mutagenesis, until a high performance variant is identified.

**Supplementary Text**

**Comparison of MLDE performance on predicting SpCas9 activity with Sg5 and Sg8 sgRNAs**

We have compared the performance of MLDE on surveying the SpCas9’s activity using data from two sgRNAs (1), Sg5 and Sg8. While using merely 10% input was sufficient to identify the three clusters of variants with high Sg5 activity (Supplementary Figure 3), using 50% input did not have high precision in predicting variants’ activities with Sg8 (Supplementary Figure 2; Supplementary Text Figure 1a). Indeed, we are confined with the overabundance of variants that showed less than 70% of the wild-type activity in the Sg8 dataset (only 10 variants showed >=70% of wild-type activity among 792 experimentally tested variants) (Supplementary Text Figure 1a). Since we used 20% of the library as the test set (136 empirical measurements for Sg8), there were only 2 variants with at least 70% wild-type activity available in the normalised test set as positive instances. In such case of rare positives, we derived extremely low precision and sensitivity from the evaluations of model performance.

When looking into the prediction of the whole library by MLDE, we merely uncovered on average two out of 10 variants that showed at least 70% of wild-type activity in the Sg8 datasets regardless of the size of input training data. The rarity of variants with >=70% of wild-type activity in the Sg8 dataset impeded what the model could learn from. In addition, Sg8 activities have a narrow range (5-95% of data range = 0.58-0.83) compared to the distribution of Sg5 activities (5-95% of data range = 0.18-0.78), that might make MLDE training difficult. Setting a floor activity threshold (we assigned -2 to the four variants with an enrichment score lower than -2) before min-max normalisation to expand the data range (5-95% of data have range = 0.29-0.71) resulted in profound improvement in precision but modest improvements in enrichment score and NDCG (Supplementary Text Figure 1a and 1b). Taken together, Sg8 is a challenging dataset for ML.

Nonetheless, MLDE exhibited surpassing performance in the prediction of the ranking of SpCas9 variants’ activities and showed success in identifying top-performing variants within the top 5%-predicted hits in the Sg8 dataset, indicated in the high scores in NDCG and enrichment (Figure 1a). When using 20% training data, 43.2% of the top 5% hits from the best MLDE prediction are also top 5% in the ground truth. With the actual experiments on variants for initial training data generation, we only needed to test 146 variants in order to identify 16 top-performing variants using MLDE, greatly reducing the experimental burden by 80% and achieving a 2.8-fold increase of resource efficiency from 0.110 (i.e., 16/146) compared to 0.039 (i.e., 37/952; 37 top 5% variants were taken from the 729 available empirical datapoints) where the full library (with 952 variants) was screened.

Whether the experimental screen could generate useful input training data for MLDE is unknown to the researcher a prior. When face with such idiosyncrasies resulting from sgRNA-specific effect, MLDE has limited usefulness in identifying variants with better performance based on an activity threshold (i.e., ≥70% wild-type). Indeed, it was also observed that some sgRNAs may be more susceptible to losing editing activity with a reducing functional dose of Cas9 (or Cas9:sgRNA molar ratio) used (2, 3). While the reasons accounting for such sgRNA-specific effect are not yet known and will need further investigation, to tackle this problem, one may test multiple conditions (i.e., more sgRNAs) and select those (such as Sg5 for SpCas9 and sg1, sg2, and s3 for KKH-SaCas9 validated in this work) that allows MLDE to generate more reliable predictions in subsequent screens.


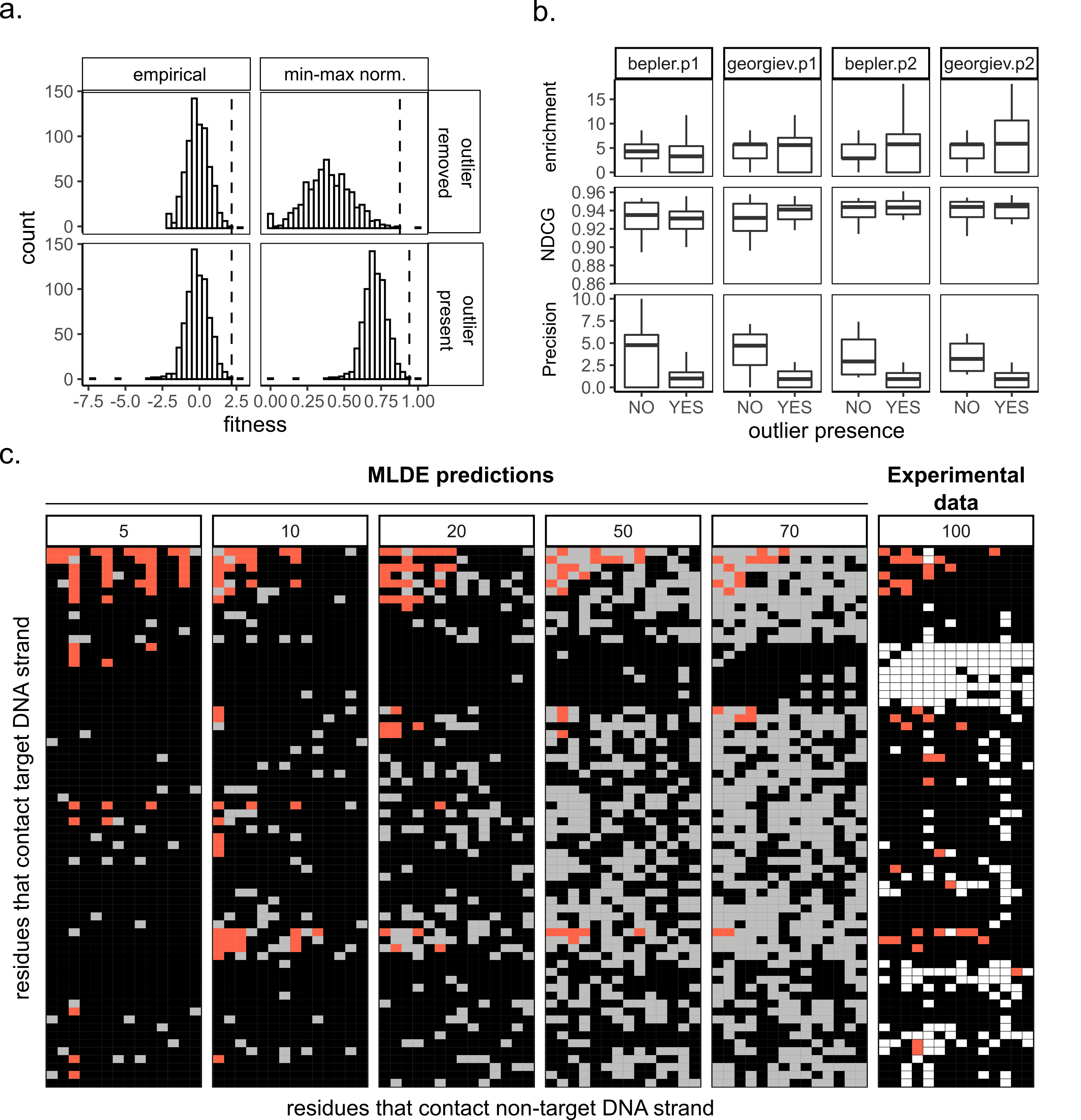
**Supplementary Text Figure 1. Performance of MLDE on SpCas9-Sg8 activity. a,** Histograms showing the distribution of empirical and normalised fitness values of the SpCas9- Sg8 activity datasets with and without outlier removal. Dash-line indicate the wild-type activity in the empirical and normalised dataset. **b,** Performance of MLDE on SpCas9-Sg8 activity after setting floor activity as -2. MLDE predictions were evaluated from 5%, 10%, 20%, 50% and 70% of input training data (n=3 independent MLDE runs for each data size). The boxplots report the enrichment, NDCG, and precision of ML on Sg8 activities using combinations of embedding (Bepler/Georgiev) and model parameters with or without removing the extremely low E-scores. The box summarizes the 25, 50 and 75 quartiles, whiskers show values within 1.5 times of interquartile ranges. **C,** Top variants identified when input of varying size are supplied to MLDE using Bepler embedding + parameter 1 settings. Top 5% variants (highlighted in tomato) identified in the best MLDE runs with varying input sample sizes that represent 5% (37), 10% (73), 20% (146), 50% (365) and 70% (510) of experimentally determined enrichment measures are shown in the tile plot. Predicted Fitness of variants present in the training data are coloured in grey. The plot at the last column (Sg8- 729 variants) showed the empirical dataset that the top 5% variants are highlight in tomato, variants with missing on-target activity information in white and variants with lower than 70% wild-type activity in black.

**Supplementary References**

1. G. C. G. Choi *et al.*, Combinatorial mutagenesis en masse optimizes the genome editing activities of SpCas9. *Nat Methods* **16**, 722-730 (2019).

2. Y. Wu *et al.*, Highly efficient therapeutic gene editing of human hematopoietic stem cells. *Nat Med* **25**, 776-783 (2019).

3. Y. Fu, J. D. Sander, D. Reyon, V. M. Cascio, J. K. Joung, Improving CRISPR-Cas nuclease specificity using truncated guide RNAs. *Nat Biotechnol* **32**, 279-284 (2014).
