# Supplementary material for: Machine learning-coupled combinatorial mutagenesis enables resource-efficient engineering of CRISPR-Cas9 genome editor activities
Source: Nat Commun. 2022 Apr 25;13:2219. doi: 10.1038/s41467-022-29874-5 (PMC9039034; doi:10.1038/s41467-022-29874-5)
Supplement: Supplementary file 2 — Reporting Summary [file 41467_2022_29874_MOESM2_ESM.pdf]

nature portfolio

Corresponding author(s): Alan S. L. Wong

Last updated by author(s): Mar 24, 2022

Reporting Summary

Nature Portfolio wishes to improve the reproducibility of the work that we publish. This form provides structure for consistency and transparency in reporting. For further information on Nature Portfolio policies, see our Editorial Policies and the Editorial Policy Checklist.

Statistics

For all statistical analyses, confirm that the following items are present in the figure legend, table legend, main text, or Methods section.

n/a	Confirmed	
☐	☒	The exact sample size (n) for each experimental group/condition, given as a discrete number and unit of measurement
☐	☒	A statement on whether measurements were taken from distinct samples or whether the same sample was measured repeatedly
☐	☒	The statistical test(s) used AND whether they are one- or two-sided<br>*Only common tests should be described solely by name; describe more complex techniques in the Methods section.*
☒	☐	A description of all covariates tested
☒	☐	A description of any assumptions or corrections, such as tests of normality and adjustment for multiple comparisons
☐	☒	A full description of the statistical parameters including central tendency (e.g. means) or other basic estimates (e.g. regression coefficient) AND variation (e.g. standard deviation) or associated estimates of uncertainty (e.g. confidence intervals)
☐	☒	For null hypothesis testing, the test statistic (e.g. F, t, r) with confidence intervals, effect sizes, degrees of freedom and P value noted<br>*Give P values as exact values whenever suitable.*
☒	☐	For Bayesian analysis, information on the choice of priors and Markov chain Monte Carlo settings
☒	☐	For hierarchical and complex designs, identification of the appropriate level for tests and full reporting of outcomes
☒	☐	Estimates of effect sizes (e.g. Cohen's d, Pearson's r), indicating how they were calculated

Our web collection on statistics for biologists contains articles on many of the points above.

Software and code

Policy information about availability of computer code

Data collection	Code used are available at https://github.com/AWHKU/RunMLDE_SpCas9.
Data analysis	FlowJo v10.7 was used to analyze data generated from flow-cytometry experiments. We use R v4.1.2 with package ggplot2 v3.3.5 and tidyverse 1.3.1 for plotting and analyses.

For manuscripts utilizing custom algorithms or software that are central to the research but not yet described in published literature, software must be made available to editors and reviewers. We strongly encourage code deposition in a community repository (e.g. GitHub). See the Nature Portfolio guidelines for submitting code & software for further information.

Data

Policy information about availability of data

All manuscripts must include a data availability statement. This statement should provide the following information, where applicable:

- Accession codes, unique identifiers, or web links for publicly available datasets
- A description of any restrictions on data availability
- For clinical datasets or third party data, please ensure that the statement adheres to our policy

Full-length amino-acid sequences of SpCas9 and SaCas9 were obtained from UniProtKB – Q99ZW2 (CAS9_STRP1) [https://www.uniprot.org/uniprot/Q99ZW2] and UniProtKB – J7RUA5 (CAS9_STAAU) [https://www.uniprot.org/uniprot/J7RUA5], respectively. The crystal structure of SaCas9 was obtained from PDB: 5CZZ [https://www.rcsb.org/structure/5CZZ]. The deep sequencing data generated in this study have been deposited in the NCBI SRA database under accession code PRJNA817034 [https://dataview.ncbi.nlm.nih.gov/object/PRJNA817034]. The GuideSeq data generated in this study have been deposited in the European Nucleotide Archive (ENA) under accession code PRJEB51773 [https://www.ebi.ac.uk/ena/browser/view/PRJEB51773]. For analysis on previously published datasets, Choi et al.'s datasets (including SpCas9 variants' activities on Sg5 on-target, Sg8 on-target, and Sg5 off-target sites) were retrieved from Supplementary Table 2 at

https://www.nature.com/articles/s41592-019-0473-0#Sec24, and Walton et al.'s datasets (including SpCas9 variants' activities on four non-canonical NGN PAMs) were retrieved from Supplementary Table S4 at https://www.science.org/doi/10.1126/science.aba8853.

Field-specific reporting

Please select the one below that is the best fit for your research. If you are not sure, read the appropriate sections before making your selection.

- [x] Life sciences
- [ ] Behavioural & social sciences
- [ ] Ecological, evolutionary & environmental sciences

For a reference copy of the document with all sections, see nature.com/documents/nr-reporting-summary-flat.pdf

Life sciences study design

All studies must disclose on these points even when the disclosure is negative.

Sample size	Sample sizes were chosen due to being able to show reproducibility and statistical significance. >300-fold more cells for lentiviral infection than the size of library being tested in genetic screens were used to ensure high fold-representation, and this was determined to be a large enough number to be representative.
Data exclusions	No data were excluded in the analysis.
Replication	All data was reliably reproduced. Methods and materials used in our experiments were described in the manuscripts to facilitate replication of our studies. The number of independent replicates for each experiment is indicated in the figure legends.
Randomization	No randomization was used for samples as samples with particular genetic constituents were needed for the experiments.
Blinding	Blinding was not relevant to the studies as samples with particular genetic constituents were needed for the experiments. Labeling of samples was used to prevent mixed up of experimental samples.

Reporting for specific materials, systems and methods

We require information from authors about some types of materials, experimental systems and methods used in many studies. Here, indicate whether each material, system or method listed is relevant to your study. If you are not sure if a list item applies to your research, read the appropriate section before selecting a response.

Materials & experimental systems

n/a	Involved in the study	
[ ]	[x]	Antibodies
[ ]	[x]	Eukaryotic cell lines
[x]	[ ]	Palaeontology and archaeology
[x]	[ ]	Animals and other organisms
[x]	[ ]	Human research participants
[x]	[ ]	Clinical data
[x]	[ ]	Dual use research of concern

Methods

n/a	Involved in the study	
[x]	[ ]	ChIP-seq
[ ]	[x]	Flow cytometry
[x]	[ ]	MRI-based neuroimaging

Antibodies

Antibodies used	Anti-SaCas9 (1:1,000, Cell Signaling #85687) and anti-GAPDH (1:5,000, Cell Signaling #2118) primary antibodies were used; HRP-linked anti-mouse IgG (1:10,000, Cell Signaling #7076) and HRP-linked anti-rabbit IgG (1:20,000, Cell Signaling #7074) secondary antibodies were used.
Validation	The use of the abovementioned antibodies are recommended by the manufacturer for applications including Western blotting. For example,<br>Anti-SaCas9 (#85687) antibody recognizes transfected levels of total SaCas9 protein.<br>https://www.cellsignal.com/products/primary-antibodies/cas9-s-aureus-antibody/85687<br>Anti-GAPDH (#2118) antibody recognizes GAPDH protein from various human cell lines.<br>https://www.cellsignal.com/products/primary-antibodies/gapdh-14c10-rabbit-mab/2118

Eukaryotic cell lines

Policy information about cell lines

Cell line source(s)	HEK293T cells are obtained from American Type Culture Collection (ATCC). MHCC97L-Luc cells are gifts from S. Ma (School of Biomedical Sciences, The University of Hong Kong). MHCC97L cells were obtained from Liver Cancer Institute of Fudan

	University RRID:CVCL_4973 (https://scicrunch.org/resolver/RRID:CVCL_4973). OVCAR8-ADR cells are gifts from T. Ochiya (Japanese National Cancer Center Research Institute, Japan).
Authentication	The identity of the OVCAR8-ADR cells was authenticated by STR profiling (Genetica DNA Laboratories). The other cell lines were not authenticated.
Mycoplasma contamination	The cells were regularly tested and showed negative for mycoplasma contamination. All cell culture medium was supplemented with antibiotic-antimycotic solution to prevent bacterial and fungal contamination.
Commonly misidentified lines (See ICLAC register)	The identity of the OVCAR8-ADR cells was confirmed by a cell line authentication test (Genetica DNA Laboratories), and is not the misidentified MCF-7/AdrR (NCI/ADR-RES) cell line.

Flow Cytometry

Plots

Confirm that:

- [x] The axis labels state the marker and fluorochrome used (e.g. CD4-FITC).
- [x] The axis scales are clearly visible. Include numbers along axes only for bottom left plot of group (a 'group' is an analysis of identical markers).
- [x] All plots are contour plots with outliers or pseudocolor plots.
- [x] A numerical value for number of cells or percentage (with statistics) is provided.

Methodology

Sample preparation	Cell cultures were treated with trypsin and diluted in complete media or PBS for flow cytometry experiments.
Instrument	BD LSRFortessaTM and ACEA NovoCyte Quanteon were used for data collection. Cell sorting was performed on a BD Influx cell sorter.
Software	All cytometry data were analyzed by FlowJo v10.7.
Cell population abundance	Drop delay was determined using BD Accudrop beads. Cells were filtered through 70μm nylon mesh filters before sorting through a 100-μm nozzle using 1.0 Drop Pure sorting mode.
Gating strategy	Viable and intact cells were gated from FSC/SSC for analysis. Within the population, infected cells that were selected for downstream analysis by gating cells expressing XFP.

- [ ] Tick this box to confirm that a figure exemplifying the gating strategy is provided in the Supplementary Information.
