# Supplementary material for: Machine learning-coupled combinatorial mutagenesis enables resource-efficient engineering of CRISPR-Cas9 genome editor activities
Source: Nat Commun. 2022 Apr 25;13:2219. doi: 10.1038/s41467-022-29874-5 (PMC9039034; doi:10.1038/s41467-022-29874-5)
Supplement: Supplementary file 3 — Description of Additional Supplementary Files [file 41467_2022_29874_MOESM3_ESM.pdf]

Title: Supplementary Data 1
Description: Number of pairwise sequences with N mismatches in the diverse dataset.

Title: Supplementary Data 2
Description: Performance of MLDE predictions on Sg5 and Sg8 on-target activities with SpCas9.

Title: Supplementary Data 3
Description: Performance of MLDE prediction on Sg5 off-target activities with SpCas9.

Title: Supplementary Data 4
Description: Performance of MLDE prediction on SpCas9's activity at NGN PAMs.

Title: Supplementary Data 5
Description: Amino acid residues selected for mutagenesis in this study.

Title: Supplementary Data 6
Description: Experimental enrichment scores determined for each KKH-SaCas9 variant with indicated sgRNAs.

Title: Supplementary Data 7
Description: Performance of MLDE predictions on KKH-saCas9's on-target activity with three sgRNAs.

Title: Supplementary Data 8
Description: Resource efficiency of identifying top-performing variants with or without applying machine learning.

Title: Supplementary Data 9
Description: List of constructs used in this work.

Title: Supplementary Data 10
Description: List of guide RNA protospacer sequences used in this study.

Supplementary Data 11
Description: List of primers and PCR conditions used for T7E1 assay.
